# The less lonely and the more satisfied you are, the merrier is … the heart!

**DOI:** 10.1093/ehjqcco/qcaf121

**Published:** 2025-09-29

**Authors:** Giuseppe Biondi-Zoccai, Mariangela Peruzzi, Giacomo Frati, Anna Sirignano

**Affiliations:** Department of Medical-Surgical Sciences and Biotechnologies, Sapienza University of Rome, Corso della Repubblica 74, 04100 Latina, Italy; Maria Cecilia Hospital, GVM Care and Research, Via Corriera 1, 48033 Cotignola, Italy; Maria Cecilia Hospital, GVM Care and Research, Via Corriera 1, 48033 Cotignola, Italy; Department of Clinical Internal, Anesthesiological and Cardiovascular Sciences, Sapienza University of Rome, Piazzale Aldo Moro 5, 00185 Rome, Italy; Department of Medical-Surgical Sciences and Biotechnologies, Sapienza University of Rome, Corso della Repubblica 74, 04100 Latina, Italy; IRCCS NEUROMED, Via Atinense 18, 86077 Pozzilli, Italy; Division of Cardiology, Santa Maria Goretti Hospital, Via Antonio Canova, 04100 Latina, Italy

**This invited editorial refers to ‘Life satisfaction as modifiable CVD prevention target: cross-cultural mediation in aging cohorts’, by C. Liu *et al*., https://doi.org/10.1093/ehjqcco/qcaf066.**

*Folks are usually about as happy as they make their minds up to be*

Abraham Lincoln

Cardiovascular practice has traditionally focused on traditional risk factors, atherothrombotic pathophysiology, cardiac remodelling, and similarly sophisticated topics, often discarding the realm of human psychology, emotions, and the brain-heart interplay.^1^ Yet, as the evidence based from high-quality studies accrue and mechanistic insights become more insightful, the heart cannot be seen any longer in clinical isolation, and the complex interaction between cardiovascular pathophysiology and psychosocial features becomes overreaching.^2^ Indeed, a recent pooled analysis published in this issue of the *Journal* and encompassing hundreds of thousands of participants, has clearly demonstrated that psychological well-being is among the key and independent predictors for incident cardiovascular disease, with population attributable fractions that rival or even occasionally eclipse some traditional factors.^3^ Strikingly, in this work by Liu and colleagues, low life satisfaction conferred a modestly but consistently increased risk of cardiovascular death, myocardial infarction, and stroke, while loneliness being a major mediating factor.

Possibly a key hurdle in facing such psychological dimensions is accurately defining them. A good pragmatic definition of loneliness for the practicing cardiologist could be that of a subjective, distressing experience that arises when the social relationships of a patient are self-perceived as insufficient, in terms of quantity, quality, or emotional depth, to meet her or his needs.^4^ Conversely, a similarly hands-on definition of life satisfaction can be the following: an individual’s cognitive evaluation of her/his overall quality of life, encompassing how well recent life circumstances align with personal values, aims, and expectations.^5^

Such associations between low life satisfaction, loneliness and clinical outcomes are not ephemeral associations but robust, reproducible patterns, observed across age strata, sexes, and continents, and, intriguingly persisted even after adjusting for other key determinants of psychosocial or cardiovascular well-being, such as smoking, depression, and sedentary lifestyles.

A puzzling question emerges, inasmuch as an elephant in a room: why has life satisfaction been so neglected as a sanctionable cardiovascular risk factor, to date?^6^ The answer, perhaps, lies in a biomedical orthodoxy that conflates objectivity with exclusion, forsaking more subjective dimensions, which are less easily measured and handled empirically, until they become statistically undeniable.^1^

Indeed, life satisfaction, though superficially intangible and often elusive, reveals itself to be anything but trivial, emerging as a formidable determinant of cardiovascular health across diverse collective settings and in individual patients as well (*Figure 1*).^7^ Most importantly, even a patient who is righteously happy today, could face in a few years a period of loneliness and life dissatisfaction, calling thus for constant vigilance.^8^ While, unlike cholesterol, blood pressure, or inflammatory markers, these psychological dimensions cannot be measured in a fool proof and mechanistic fashion, their predictive strength may informatively complement the prognostic accuracy of the most established clinical biomarkers.^9^ Furthermore, we should not simply consider joy of life as *the primum movens* of such psychological well-being. Rather, a profound sense of agency, coherence, and self groundedness give impact and meaning to the lives of our patients.^10^ Accordingly, an unsatisfied life adversely impact cardiovascular physiology, silently yet steadily, through insidious mechanisms involving cortisol surges, inflammatory cascades, and behavioural disengagement. In younger individuals, life dissatisfaction may impact on survival and quality of life more aggressively, compounded by instability, underachievement, and the turbulence of modern adulthood.^11^ Contrastingly, loneliness, which is often mistaken for solitude, lurks even in crowded rooms and busy cities, hiding behind smiles and surface chatter, especially among the elderly or the most vulnerable ones, exerting a slow but ruthless pressure on the cardiovascular system, dysregulating the autonomic nervous system, distorting sleep architecture, and fostering a biologic milieu primed for cardiovascular injury. While both constructs may appear challenging to grasp and act upon, bordering subjectivity, their physiological echoes impact on several key cardiovascular players such as endothelium, platelet function, and arrhythmic risk.^12^

**Figure 1:**
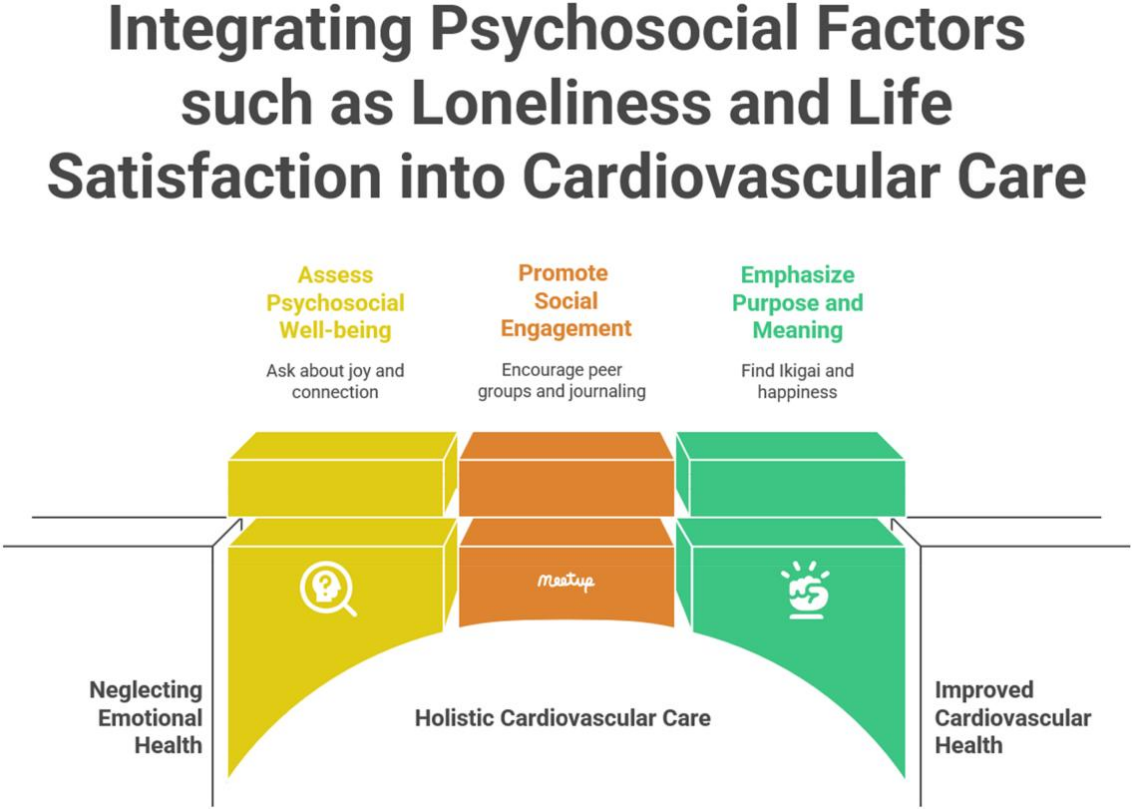
Overcoming psychosocial barriers to cardiovascular well-being, focusing on loneliness and life satisfaction.

When the apparently intangible burdens of loneliness and life dissatisfaction settle upon a patient, the heart, not merely metaphorically, bears the cost, as clearly demonstrating by an expanding epidemiological evidence base, which unveils a consistent, sobering portrait: psychosocial malaise elevates the risk of several cardiovascular outcomes, as well impairs quality of life and adherence to life saving interventions, often insidiously.^13^ On top autonomic dysregulation and systemic inflammation, endocrine dysfunction also play a key pathophysiologic role.

Despite the strengths of the work by Liu *et al*., several caveats should be borne in mind, calling for interpretive caution. In particular, measurement issues should not be discounted, as loneliness and life satisfaction are self-reported constructs, prone to semantic drift across languages and cultural frames, over-reporting, and so forth.^14^ Whenever a novel or neglected risk factor or pathophysiologic mechanism is purportedly showcased, there is also a looming risk of type I error. Moreover, the fleeting nature of affective states juxtaposed with the chronicity of cardiovascular pathophysiology does not imply temporal stability or constant biological relevance. As usual in epidemiologic studies, association cannot qualify *per se* for causation, and thus further mechanistic research is welcome.

Irrespectively, psychosocial well-being, once relegated to the margins of cardiovascular risk discussions, now commands serious clinical attention, a shift catalysed not by sentiment but by statistics.^15^ Indeed, to ignore loneliness or life dissatisfaction is to neglect a pathology as real as atherosclerosis, albeit more insidious in its presentation. What should a physician do, then, to gauge and address these additional clinical dimensions? It boils down to a few simple questions: not only how are you, but who are you seeing, what brings you joy, and when did you last laugh out loud? Screening tools are fine, but even a 30-second inquiry delivered with authenticity may carry substantial diagnostic weight. Educational, social, and psychological support are crucial and should be routinely implemented whenever appropriate and feasible, with the hope that resources dedicated to them will expand in the future. As for patients themselves, they are not without recourse: structured journaling, peer-led circles, the comfort of key books such as *Ikigai: The Japanese Secret to a Long and Happy Life* or *The Happiness Advantage* may, in their modesty, prove meaningfully beneficial. To be frank, we also remain quite positive that taking care of our patients’ sense of belonging and life satisfaction can benefit also us as individuals and caregivers, bringing us as well professional and personal benefits, which may extend to our dear ones.

## Data Availability

No original data were used to generate this manuscript.
